# A Sociological Analysis and Exploration of Factors Associated with Commercial Preparations of Smokeless Tobacco Use in Sri Lanka

**DOI:** 10.31557/APJCP.2021.22.6.1753

**Published:** 2021-06

**Authors:** Mohamed Mahees, Hemantha Kumara Amarasinghe, Udaya Usgodaarachchi, Nilantha Ratnayake, W M Tilakaratne, Suresh Shanmuganathen, Sajeeva Ranaweera, Palitha Abeykoon

**Affiliations:** 1 *Department of Sociology, Faculty of Arts, University of Colombo, Sri Lanka. *; 2 *Family Health Bureau, Ministry of Health, Colombo, Sri Lanka. *; 3 *National Cancer Control Programme, Ministry of Health and Indigenous Medicine, Narahenpita, Colombo, Sri Lanka. *; 4 *Office of the Provincial Director of Health Services, Western province, Maligawatte, Colombo 11, Sri Lanka. *; 5 *Department of Oral and Maxillofacial Clinical Sciences, Faculty of Dentistry, University of Ma-laya, Malaysia. *; 6 *OMF Unit, General Hospital, Kalutara. *; 7 *Expert Committee on Tobacco, Alcohol and Illicit drugs, Sri Lanka Medical Association, Sri Lanka. *; 8 *World Health Organization, Country Office, Sri Lanka. *

**Keywords:** Smokeless tobacco, commercial preparations of smokeless tobacco, betel chewing, focus group discussion

## Abstract

**Background:**

Use and addiction to commercial preparation of Smokeless Tobacco (CPSLT) is creating new socio-cultural issues and health challenges in Sri Lanka. The objective of this sociological study is to investigate and analyse the socio-cultural factors that influence CPSLT use Sri Lanka to enable development of effective interventions.

**Methods:**

This is a qualitative study for which data was collected through in-depth interviews in selected groups that use CPSLT. Thirty-five interviews were carried out in seven of the 24 districts in the country representing urban, rural, plantation communities and different livelihood and ethnic, gender and age groups in the year 2016. Purposive and snowball sampling techniques were used for selecting interviewees. The data was analysed by using qualitative data analysis techniques and sociological perspectives.

**Results:**

This study reveals that the CPSLT use has integrated with the culture of several sociological and livelihood groups. Products such Thool (tobacco powder) and Maawa (dried areca-nut with flaked tobacco and some flavoured chemicals) were identified as the most popular forms of CPSLT. Use of CPSLT has developed as a silent sub-culture specific to several social and livelihood groups. The informal CPSLT industry operating in the urban and sub-urban areas is influencing the school children and youth engaged in sports. Different groups of users express different reasons and justifications for its use.

**Conclusion:**

Use of CPSLT is closely integrated with the day to-day lifestyle and values of people of specific groups. and is an unseen part of life. Therefore, interventions will be urgently required to control the use of CPSLT to prevent its significant health impacts, considering the different contexts, symbolisms and justifications of its among the different groups.

## Introduction

Smokeless Tobacco (SLT) in the form of betel chewing is a deeply ingrained lifestyle habit in Sri Lanka especially in the villages and estate sector labour communities. Ingredients used for betel chewing such as betel leaf, tobacco, areca-nut and lime are available in the open market or is home grown. No taxes are imposed at any point in the supply chain. Commercial Preparations Smokeless Tobacco (CPSLT) are also becoming popular mostly in the younger generation in Sri Lanka in Urban and Semi-urban communities. CPSLTs namely Gutka, Panparag, Hans and Maawa which are imported from neighbouring countries. Some products identified as Babul and Beeda are produced in Sri Lanka (Somatunga et al., 2012). However, commercial preparations are not available in the open market and are being sold in the black market only to the known users. 

Knowledge and attitude on SLT and areca nut control was assessed among the nursing students in the central province of Sri Lanka, revealed that 92% agreed upon the fact that SLT use is increasing in popularity among youth and adolescents (Hettiarachchi et al., 2020).

World Health Organization (WHO) STEP wise approach to chronic disease risk factor surveillance (STEPS) Survey of 2015 showed that 29.4% males and 0.1% females were current smokers in Sri Lanka, while 26% of males and 5% of females were current users of SLT (Ministry of Health Sri Lanka, 2015). The WHO Global Youth Tobacco Survey (GYTS) 2015 showed that 3.2% of boys and 0.2% of girls between 13-15 years of age smoked at least once during the 30 days preceding the survey in Sri Lanka. The prevalence of SLT use among students of this age-group was 4.2% among boys and 0.5% among girls (World Health Organization, 2015, World Health Organization US Centers for Disease Control and Prevention, 2015). A study conducted among those over 30 years of age in the villages and estates of the Sabaragamuwa Province of Sri Lanka showed that 53.7% of the study population chewed betel daily and 27% of them were ever smokers (Amarasinghe et al., 2010).

Programme evaluation study conducted among 663 dental surgeons in Sri Lanka, showed 20.1% of them consumed tobacco in any form in the past and 2.1% used SLT and 2.3% consumed areca nut (Jayasinghe et al., 2021). 

Health hazards of SLT are well documented. South East Asia is experiencing the highest burden of its related co-morbidities. Among them, cancers of the lip, oral cavity and pharynx are highly devastating diseases resulting from the use of SLT. According to the Globocan 2020, 377713 new cases of lip and oral cavity cancers and 177757 deaths were reported which ranked at 16th among all cancers (Ferlay etal., 2020). The incidence of cancer of the oral cavity ( ICD 10: C00-C06) in Sri Lanka, standardized to the world standard population in the year 2014, was 15.6 and 3.7 per 100,000 populations, in males and females respectively (National Cancer Control Programme, 2014). 

It is well known that the use of SLT products is a culturally bound habit in Sri Lanka where betel-quid chewing has been practiced for over 2000 years (International Agency on Research on Cancer., 2004). Moreover, as the CPSLT are still not well accepted as compared to the traditional betel-quid chewing, it is practised as a concealed behaviour. According to the hospital reports, it is observed that oral potentially malignant disorders (OPMD) is increasing in youth, and most of them are using CPSLT products.

Since this trend of SLT usage in Sri Lanka is posing a new set of health problems, it is important to develop and implement effective interventions to control its usage. However, the nature of concealed behaviour on SLT usage, and the lack of published information on the determinants of such use has hampered the collection of relevant information through quantitative questionnaire-based population surveys. All available studies on SLT use in Sri Lanka only gives basic information on the prevalence and gender difference, but no information is available on underlying socio-cultural factors and other determinants of use. The aim of this sociological study is to investigate and analyse the socio-cultural factors that influence the CPSLT use in Sri Lanka. This study collected basic quantitative data on socio-demographic distributions using existing island-wide survey reports and applied a qualitative approach to understand the underlying and determinant factors for CSLT use. 

## Materials and Methods

This is an exploratory qualitative study based on the approach of examination of determinants of CPSLT use among different livelihood groups in rural, urban and estate communities and its impact on their personal lives, family and community. 

The in-depth interview method was used to data collection technique to gather qualitative aspects of socio-cultural, class, ethnic, livelihood and political aspects of CPSLT users. In-depth interviewer guide was prepared in English and translated to Tamil and Sinhala by the expert team of Sociologist, Senior lecturer in Sociology, medical officers and Consultant in Community Dentistry. 

Thirty-five (35) in-depth interviews were carried out in the districts of Colombo, Gampaha, Kalutara, Nuwara-Eliya, Kurunegala, Pullam, Anuradapura in the year 2016 based on the purposive sampling and snowball sampling techniques. Above districts were randomly selected from all district based on the availability of particular high-risk group. The respondents were identified with the help of Public Health Inspectors attached to Ministry of Health and Police Officers of the areas. The three-wheel taxi drivers of the relevant areas assisted in the first sample of snowball techniques. The sample of 35 respondents represented different livelihood groups (transport workers, garment factory, plantation community, urban low-income groups, fishermen, pavement hawkers, self-employed, private and government sector employees) and ethnic groups. The different social-geographical components of rural, urban and estate communities were also included. 

The data was qualitatively analysed based on the sociological themes and theories.

*Ethics*

The required ethical considerations were incorporated into the study and ethical approval was obtained from Ethical Committee of the Faculty of Medicine, University of Colombo, Sri Lanka. 

*Funding*

National authority on Tobacco and Alcohol, Sri Lanka was conducted this sociological study by obtaining a grant from Campaign for Tobacco Free Kids, fund allocation number: Sri Lanka G 84.

*Data sharing statement*

Data is available by emailing to the corresponding author: hemanthaamarasinghe @yahoo.com.

*Patients and Public involvement statement*

The summary results of this study were discussed conducted at the ‘World No Tobacco Day’ commemoration on 30th May 2018 and 2019. Workshops were conducted and disseminated the findings of the study to the Public Health Inspectors who are working in the grass root level.

**Figure 1:**
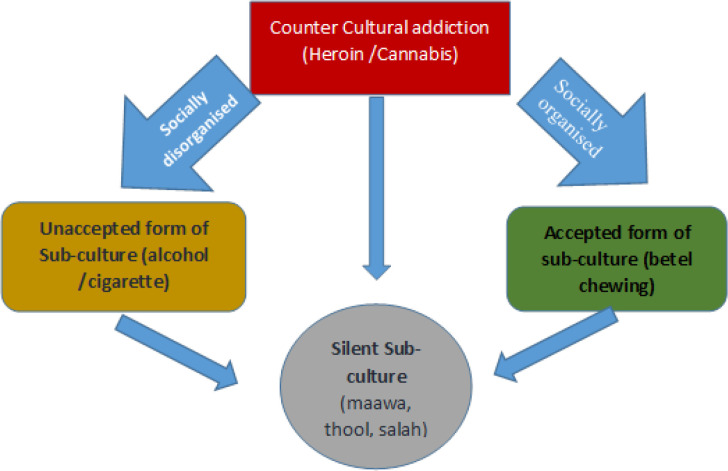
The Silent Sub-Culture ofSmokeless Tobacco

## Results

*Types of CPSLT*

Amarasinghe et al., (2018) has identified some of the smokeless tobacco use and its impact in the Sri Lankan society. However, this qualitative study attempts to recognize some forms of smokeless tobacco varieties depending on the regional business trade names and local terms given by the community. The following CPSLT varieties were identified by the study. 

*CPSLT : Snuff / Thool / Dappi*

The snuff is popularly known as thool or dappi or Mukkupodi. Thool is a Tamil word which refers to power or dust; here the tobacco powder is called as thool. According to Somathunga et al., (2012), thool can be called as red tooth powder or tobacco powder which is imported from India or locally prepared. It is powdery preparation of tobacco and it is used by placing in between the teeth and gums. They consider thool as a medicine and it has been socialized as a cultural practice among them. The thool was mainly used by elders and adults and not youth or children in the past. 

*CPSLT: Maawa*

Maawa is also known as pani puwak, sweet, ash or sometimes pan parag depending on the local culture and identification of commercial brand by the users in different parts of the country. However, maawa is the popular term used in many parts of the country. Maawa is prepared with pieces of shavings of dried areca-nut with flakes of tobacco, slaked lime and some honey or any sweet chemicals. It is chewed and kept in the mouth specially behind the lips or in between teeth and gums. 

Maawa is also produced and packed in different ways and sold at different prices. There are also imported maawa from India but most of the maawa commercial preparations are locally produced. It was revealed that most of the maawa commercial preparations are added with some honey or special flavor called Kashmeer imported from India. Many persons have chosen to use maawa instead of cigarettes According to the in-depth interviews carried out in different parts of the country, it was revealed that maawa is good to use due to: (1). Smoking being controlled by many rules, (2). Public humiliation (3). Explicit negative health implications. However, maawa use is more cultural than economic. 

*Adaptive Addiction to CPSLT and Salah*

Salah is another CPSLT identified by this study. It was found only from Colombo and Nuwara-Eliya. Sometimes, it is very difficult to separately identify Salah from baabul or maawa. Salah is supposed to be originated from babul, beeda (which is made of tobacco leaf, areca-nut, betel leaves and lime) but salah is free from betel leaves. Salah is prepared with sliced areca-nut, honey mixed tobacco and substance called kashmeer. Salah is usually kept behind the lips or in between teeth and the gum. 

It is also doubtful whether salah and pan parag, which has been identified in other studies (Somatunga et al., 2012) are same commercial preparation used as smokeless tobacco. However, according to any of the interviews carried out under the sociological analysis, nothing was found equal to pan parag from any of the fields. 

Salah is not preferred by all like maawa because it causes injuries to the mouth and causing difficulty in chewing food. It is only hard-core SLT users or most addicted users prefer salah considering the strength and intoxication of salah. There are many locally produced tobacco substances such as kalu or Sinhala dunkola. What is most crucial is that there are many varieties of locally produced CPSLT items which are called by regional specific names with slightly different ingredients. 

Salah is the adaptation of CPSLT used in the urban areas. The internal community or social control mechanism is the most influential way to control these practices by minimizing the adaptive use of CPSLT. The CPSLT users adapt to new tobacco commercial preparations as a result of rules and pressure from the tobacco controlling agencies.

*Perception on use of CPSLT*

The perception about the CPSLT is determined by demographic, socio-cultural and regional factors. The perception is influenced by both psychological and sociological factors. CPSLT has been the common platform through which a peer socialization smoothly take place without serious resistance and as a result, youth peer groups are easily and positively influenced by CPSLT. Social interaction, peer solidarity, social approval, and substance use, work together as positive reinforces to sustain drug use (Akers, 1992). 

The most serious challenges of controlling CPSLT such as maawa, baabul and thool depend on changing the perception of those substance users. This study reveals that the perception of CPSLT is always either socially or culturally created. As Burger (1976) argued that the reality is socially constructed. The perceived health risk associated with CPSLT is not properly understood nor can tobacco users be convinced of them. According to the results of the interviews persons who are badly addicted to CPSLT have very poor educational background and no critical perception about the CPSLT and its negative impact. Some CPSLT users believe that the substance function as informal local drugs for some disease such as headache and phlegm disease. 

“I have been using thool for more than 20 years. Nothing wrong happened to me so far. If thool causes cancer I would have died by now” (Anuradhapura)

*Power Relationship behind CPSLT*

The power relationship matters a lot in terms of producing, distributing and selling the CPSLT and the political economy of the area directly or indirectly get involved with protecting the business zone and trade network. There is a well-connected and protected customer base for CPSLT business. It is far more important to comprehend the black economy in association with CPSLT business and the informal socio-economic mechanism functioning within the formal economic activities. Although it is difficult to provide clear evidence that the CPSLT business chain is having no link with hidden political economy of the urban areas, there can be economically motivated power groups behind this business chain. This situation can be well understood by the theory of soft state introduced by by Myrdal (1970) who argues that although there are rules and regulations to control some unwanted things in developing countries, the law breakers strategically find the lope-holes in the law and order and engage in anti-social activities such as CPSLT. 

The practice of CPSLT is also known to be a powerful social action found among local power context. According to the in-depth interviews, it is only the socially or culturally dominant groups are aware of many of the tobacco products which are used as CPSLT. Majority of the respondents were of the view that CPSLT can be controlled with the help of social power groups in the area. The community leaders, religious centres, schools, women and community-based organizations can play a leading role in controlling CPSLT. The following two statements from two respondents clearly indicate the power of local set up and its possibility of controlling CPSLT through the social power. 

“It is only after the new inspector came here, I stopped using baabul and maawa. He raided all the maawa sellers and arrested the maawa users” (Kurunegala) “The priest of the church knows about CPSLT and he preached not to get addicted to them and seriously advised the sellers to stop it at the Sunday Service of Church” (Gampaha)

Except in Colombo and Kalutara, all other study areas (Anuradapura, Gampaha, Nuwara-eliya, Kurunegala and Putlam), the respondents stated that the religious priests or religion can control the SLT practice to a greater extend by using the informal social power of priests. The religion (Temple, Church, Mosque) can play a central role in between three stake holders (MOH, Police and community). Since CPSLT is subjectively connected with livelihood and lifestyle of people as an integral part of culture which functions through silent social currents. 

*Silent Sub-culture and CPSLT*

CPSLT users identified themselves as a group of persons belong to a specific lifestyle and certain norms and fashion in terms of using CPSLT such as maawa, baabul, thool and other items. In addition to the concepts of main culture and sub-culture, the third concept is “counter culture” which refers to total violation of main culture and adherence of counter values and life style (Haralambos and Holborn, 2009). According to this study serious addiction to heroin and cannabis is a counterculture. On the other hand, betel chewing belongs to main culture of majority of people because it is part and partial of culture of Sri Lanka. Although betel chewing is found to be medically harmful to oral health (Amarasinghe et al., 2010), it has become part of culture and a mode of sharing social relationship. 

Even if cigarette and alcohol just as betel chewing are in the context of main culture with or without social protest, the CPSLT such maawa, salah and thool are still in the silent mode of sub-culture. According to the analysis of this study, CPSLT can be considered as “Silent Subculture” found among specific social groups living in specific environmental or social context (Figure 1). This study reveals the new form of silent sub-culture of using CPSLT which would challenge all other forms of sub-culture or general mode of cultural practices in association with tobacco and alcohol use in Sri Lanka.

*CPSLT and Sports*

Sociologists study the sports analogy and drug use and how myths are associated with sports. The corporate or organizational deviance in sport is now recognized as a serious problem. The deviant behavior in sports is mostly identified in terms of use of drugs and substances (Corner, 2009). The high school athletes who play sports smoke tobacco products at a lower rate than non-athletes, but use CPSLT at a higher rate, according to a study published by the Centers for Disease Control and Prevention in today’s Morbidity and Mortality Weekly Report (MMWR) (Center for disease control and prevention, 2015).

 The sociological study carried in several districts in terms of CPSLT clearly indicates that sports like rugger, football and cricket are influenced by CPSLT. It was found that sports or group game venues have been a kind of social access for boys to get used to CPSLT like maawa. Boys who play rugger or football in Colombo, Negambo and Chilaw area have addicted to maawa. Boys who do not play for the team also come to ground or club in the evening and have companionship with their friends. Apparently, this is the time that they use to take most of the CPSLT. Sometimes some boys take cannabis too on these occasions. These two statements made by a rugby player is important to understand the correlation between rugger and maawa. 

“In the rugger game, we need to run throughout the game everywhere in the ground and it is a very hardworking game, so eating maawa during the game give us additional energy and it motivate us to do anything irrespective of tiredness (Colombo) “We all boys get together in the evening at the ground and take maawa. We are not suspected by anyone because we go there to play football (Gampaha).

The qualitative field data reveal that there are four major macro-level factors such as (1) Livelihood (2) School and sports (3) Family and neighbourhood (4) Psychological conditions. It was revealed that school and sports are most influential factors that make the background for learning and addiction of CPSLT. The school and sports are closely associated with peer culture and socialization. The use of CPSLT practice is rapidly spreading among youth who engage in group games. According to the findings, sports activities appeared to have justified use of CPSLT during and after the game, it can be called as ‘Rationalization of CPSLT use in sports’. 

Although CPSLT is being criticized on medical grounds, when it comes to the real ground realities of the youth culture specifically based on sports, CPSLT is a matter of solution for them and they consider maawa and thool as the best mood elevating substances which save them from other extremely dangerous drugs. According to boys who engage in sports and others who generally use CPSLT state that maawa and thool are the best substitutes for cigarettes, alcohol, cannabis and heroin. On the other hand, when a person starts his drug addiction with maawa, it will also take him to other higher and extreme levels of substance addiction. Thus, maawa functions as upward and downward trend in its ladder. The CPSLT such as maawa is powerful enough to function in between two addicted substance dynamics. According to the interviews, some boys who have started with maawa ended their addiction with cannabis or heroin. On the other hand, some boys have found the way out from the serious drug or alcohol addiction by using the maawa. 

*Youth fashion, music, sex and CPSLT*

Use of thool, salah, maawa and other CPSLT are well integrated with youth culture such as fashion, music and premarital sex. It was well identified that young boys who behave in rather deviant manner by recognizing them as a ‘gang’ of the area in Chilaw, Negambo, Anuradapura These boys are interested in tattoo culture and most of the boys who are addicted to maawa had tattoo marks on their body. In addition to tattoo culture, there was a close relationship between maawa and thool users and pigeon keeping which are urban slum culture as well as a source of income. 

In most of the urban and sub-urban areas of Sri Lanka, the open musical shows are organized three to four times a year. The boys including garment factory workers are used to go for these musical shows and entertain themselves by dancing while songs are sung by popular artists on the stage. According to the findings of in-depth interviews, most of the boys used to take CPSLT before going to the musical shows and they share those tobacco products with their friends. In addition to this practice, boys who were interviewed as respondents from Anuradapura, Kurunegala and Gampaha mentioned that they take maawa before they go to boxes in the film theatre with their girlfriends. These respondents further mentioned that they use maawa when they go to a hotel room with their girlfriend. They believe that maawa gives them a kind of additional sexual motivation and they can spend more time for sex with their girlfriend. According to the results of in-depth interviews, maawa is a kind of vicious circle for youth 

*CPSLT and Education*

This is the most alarming situation of CPSLT use in Sri Lanka. When the school becomes the socialization agency of CPSLT such as maawa and thool, it is very difficult to control CPSLT in Sri Lanka. According to the in-depth interview, in all the districts, children have the greater chance of learning this behavior. As described by sociologists, social learning of deviant behavior promotes anti-social and drug addicted behaviour (Gidden, 1989). Most of the interviewed respondents mentioned that they learned to use CPSLT first in the school through their friends. 

“We started eating maawa from the school. Our gang did not care about the persons who did not eat; those who did not eat did only studies” (Colombo). “Ungi and Mahesh aiya gave us maawa, we ate them in the school toilet during the interval, we wrote our exam papers after eating maawa because then we feel easy to write” (Anuradapura) “I first used thool in the school taking from my friends” (Kurunegala)

According to the above statements of respondents, it is clear that the CPSLT has badly influenced the schools and children are being socialized to CPSLT use by the informal and hidden CPSLT industry which always targets the most vulnerable group of schooling children.

*CPSLT and Livelihood and Gender*

The study identified several livelihood groups which are badly affected by CPSLT. The livelihood groups such as three-wheel drivers, security men, daily earners, porters, garment factory workers, construction workers, drivers and conductors in transport need to be separately studied by researches in order to have more subjective and qualitative information on different aspects of CPSLT use in Sri Lanka. 

The socio-cultural difference between man and women is generally known to be gender and gender is a cultural product which influences many of the human behavior. If the CPSLT use in Sri Lanka is analyzed through gender perspective, it is possible to witness very clear different forms of behavior in association with the use of CPSLT. It was revealed that women hardly use heroin or cannabis and they are also less likely to use alcohol compared to men. However, when it comes to CPSLT, the trend of using CPSLT by women is comparatively significant and high. In addition to betel chewing practice, Muslim and Tamil women have got addicted to thool and some women in the fishery and plantation sector are addicted to maawa. It is the middle age married women mostly got affected to this practice. Women in the Tamil speaking community have become very much vulnerable in using thool and they are totally unaware of the health issues related to CPSLT due to language and other communication limitations.

In conclusion, this sociological study focuses on CPSLT except the betel chewing in Sri Lanka. The study was totally based on the qualitative data collection and qualitative analysis of factors and forces behind the use of CPSLT. This sociological explorative inquiry studied the subjective meanings and experiences of CPSLT users in different socio-economic contexts. The CPSLT locally known as thool and maawa are more popular and spread all over the country. The CPSLT has become an integral part of Sri Lankan culture and the degree of addiction to CPSLT depends on the specificity of the sub-culture. The youth sub-culture in association with sports and fashion lay the foundation for the use of CPSLT. The CPSLT addiction is very serious in the livelihood groups such as transport workers, three-wheel riders, construction workers, fishermen and plantation workers. The CPSLT has gained control over the religiously valued culture and become a very normal practice among Muslims and Plantation community. Moreover, the informal urban industry which promotes CPSLT among school children and youth sports clubs is becoming a powerful economic and political mechanism.

*General recommendations*

As it was clearly pointed out and discussed CPSLT is hidden and silent cultural phenomenon, it must be controlled mainly through the knowledge and awareness with maximum precautious measures. Educating school children against CPSLT through art exhibition, drama and by other possible performing arts methods will be immensely useful. The program connected with tobacco victim group developed by the National Authority on Tobacco and Alcohol and Apeksha hospital, Maharagama which is called “Voice of Blue Pea” will be another strategy and VOBP movement could be advocate to the youth, soccer and rugger teams. Since most affected of CPSLT coming different low-income groups, formation of anti-CPSLT campaign must be carried out with the special support of three-wheel riders. All the public awareness campaign and officers such PHIs engaged in these movements should be awarded with some rewards. The informal social mechanism such Temple, Church, Mosque that hold the control of urban and rural community must play leading role in education general public on the harm of CPSLT. All the programs and campaigns must also be done in Tamil so that Tamil speaking community who have become victim of CPSLT. It would be practically necessary to introduce some alternative and no harm substitute for maawa and thool in order to save the addicted people specially in the sectors of construction, plantation and factory works.

## Author Contribution Statement

All authors designed the study, generated hypotheses, interpreted the data and critically reviewed the manuscript. MM analysed the data and wrote the first draft, HA, UU and NR contributed in data collecting and writing and editing the manuscript, WT, SS, SR and PA advised on study design and edited the manuscript. All authors approved the final version.
